# A Retrospective Analysis of Hospital Electrocardiogram Auto-Populated QT Interval Calculation

**DOI:** 10.7759/cureus.9317

**Published:** 2020-07-21

**Authors:** Adam L Rosenblum, Ariana C Dremonas, Scott C Stockholm, Nicholas L Biondi

**Affiliations:** 1 Internal Medicine, Cape Fear Valley Health System, Fayetteville, USA; 2 Internal Medicine, Campbell University School of Osteopathic Medicine, Buies Creek, USA

**Keywords:** qt interval, qt calculation, 12-lead, electrocardiogram (ecg/ekg), torsades de pointes (tdp)

## Abstract

Background

The current electrocardiogram (ECG) standard for rate correction of the QT interval (QTc) is a power function known as the Bazett formula (QTcB). QTc formulae are either power functions or linear functions. QTcB is known to lack reliability, as heart rate (HR) rises from or falls below 60 beats per minute (bpm). The American Heart Association (AHA), the American College of Cardiology Foundation (ACCF), and the Heart Rhythm Society (HRS) have recommended using other formulae in place of QTcB since 2009. The Epic Electronic Health Record System (Epic Systems Corporation, Verona, WI) automatically populates the Fridericia formula (QTcFri) on hospital ECG reports without any provider calculation.

Methods

We aimed to retrospectively investigate the effect of QTcFri on one year of ECGs in the Epic Electronic Health Record (EHR) at a single tertiary care center. Inclusion criteria for ECG reports specified HR 60-120 bpm without QRS duration > 120 ms. Gathered data from Epic EHR ECG reports included patient age, sex, HR, QRS duration (QRSd), QT interval, QTcB, and QTcFri. EHR documented 61,946 ECG reports for the year, with 44,566 meeting criteria for inclusion. General statistical methods included range, median, mean, and standard deviation. Confidence intervals were assessed to maintain the fidelity of analysis. The normality of data distribution was assessed with Kolmogorov-Smirnov testing. The Wilcoxon rank-sum test was then performed to confirm a statistically significant difference between the Bazett and Fridericia formulae. The ∆QTc analysis was conducted on prolonged QTc (males > 450 ms; females > 460 ms) and severely prolonged QTc > 500 ms data subsets. A value of p<0.05 was interpreted as significant. Statistical analysis was performed using SPSS statistical software (IBM Statistics, v. 26; IBM Corp, Armonk, NY).

Results

The 44,566 ECG reports demonstrated 57% female gender and a mean age of 57 ± 17.5 years. The mean HR was 83 ± 14.7 bpm and the mean ∆QTc was 23 ± 12.9 ms shorter with QTcFri. Mean data showed minimal variation between sexes: age, heart rate, uncorrected QT, QTcB, QTcFri, and ∆QTc varied by less than 2%. Mean QRS varied by 4% between sexes. The Wilcoxon rank-sum test revealed 44,127 ranks with a negative difference, 0 ranks with a positive difference, and 439 ties, p <0.001 (99% CI: 22.5 ms, 23.0 ms). QTcB identified 37.4% (16665/44566) ECGs prolonged. Using QTcFri, 21% (9371/44566) of the total ECGs corrected to normal QTc (<450 ms (men) and 460 ms (women)). QTcFri use reduced the number of ECG reports with QTc > 500 ms by 57.3%. A total of 125 ECG reports, 117 females and eight males, corrected to normal gender-specific QTc with QTcFri. The mean decrease in QTc with the Fridericia formula when QTcB > 500 ms was 31 ± 14.5 ms (99% CI: 30.4 ms, 31.7 ms).

Conclusion

Our data from the Wilcoxon rank-sum analysis indicated that the EHR QTcFri analysis yields a statistically significant difference (p < 0.001) in QTc calculation of 22 ms over 44,566 ECG reports. The data showed a 21% reduction in inaccurately documented test results. The utilization of this resource will provide the most accurate and clinically relevant data to inform clinical decision-making. Accurate QT interval calculation will better inform downstream clinical decision-making through a wider scope of therapeutic intervention. This analysis is readily available to clinicians without calculation and its awareness will benefit patient care.

## Introduction

The QT interval of an electrocardiogram (ECG) was originally named by Willem Einthoven in 1895 in accordance with the mathematical tradition established by Descartes [1]. The interval corresponds to the electrical systole of the cardiac cycle - the time duration spanning from the opening of fast Na^+^ and L-type Ca^++^ ion channels, initiating Phase 0 of ventricular depolarization, until the closing of inward-rectifier K_1_ current dependent channels, terminating phase 4 ventricular repolarization. The magnitude of the interval is inversely proportional to the heart rate (HR): increasing or decreasing heart rate will respectively contract or expand the time interval between beats, affecting the QT interval on ECG.

Dr. Henry Cuthbert Bazett evaluated the ECGs of 39 people in 1920 to derive the current standard QT interval rate-correction (QTc) formula, used as a surrogate evaluation of mechanical systole [2]. Since Bazett’s analysis, numerous approaches with either power functions or linear functions, each with inherent idiosyncrasies, have been proposed. Among these formulaic curiosities is a consistent observation that Bazett’s calculation (QTcB) produces increasing QTc inaccuracy, as the heart rate rises in excess or falls below a rate of 60 beats per minute (bpm) [3].

Evidence collected since the 1980s prompted the American Heart Association (AHA), the American College of Cardiology Foundation (ACCF), and the Heart Rhythm Society (HRS) to issue guidelines on appropriate ECG interpretation in 2009, recommending against the use power-function formulae [3]. The unreliability of QTcB at HR above or below 60 bpm continued to be corroborated in research following the 2009 Guidelines, and the recommendation for alternative formula use over QTcB was reaffirmed by the 2017 updated AHA Scientific Statement [4]. 

One of the greatest barriers to changing the ECG standard from QTcB to another QTc formula has been the lack of consensus evidence regarding which alternate formula demonstrates clinical superiority. Retrospective research from 2016 demonstrated that the Framingham (QTcFra) and Fridericia (QTcFri) formulae exhibit superiority to the Bazett Formula at heart rates less than 90 beats per minute [5]. QTcFra outperformed the Hodges formula (QTcH), the Rautaharju formula (QTcR), QTcB, and QTcFri with regard to both 30-day and one-year mortality - however, no statistically significant distinction between QTcFra and QTcFri was determined for either metric [5]. Rautaharju formula (QTcR), a linear-function formula, was studied in 57,595 subjects and is specific to both gender and QRS duration [2,6]. Currently, no prospective data exist to corroborate these retrospective analyses and identify which formula is to replace QTcB. Guidelines continue to recommend a specific methodology over a specific formula: linear-function formulae (i.e. QTcH, QTcR) use is supported by computational model evidence over power-function formulae use (i.e. QTcB, QTcFri, QTcFra) [2-3]. The guidelines further recommend formula citation with each interpretation in an effort to avoid confusion between ECG documented QTcB and any other corrected QT formula [3].

Accurate QTc analysis is vitally important to patient care. QTc use is integrated into the clinical decision-making process for both drug class and frequency of medication administration - due to the common off-target side effect of many medications to prolong the QT interval. Additionally, using more accurate QTc calculation is essential for monitoring the risk of ventricular arrhythmia e.g. Torsades de Pointes (TdP). Normal QTc is gender-dependent, with male values ranging from 350-450 ms and female values ranging from 350-460 ms [3]. The risk for TdP increases as QTcB increases at values greater than 500 ms [7-8]. QT interval prolonging medications are normally held as QTcB approaches 500 ms - a formula-dependent threshold. Increasing provider awareness of available QTc methods has the potential to directly impact patient care via decreasing the above metrics by using guideline-based formulae. 

## Materials and methods

Study design

This single-center, retrospective, non-randomized, non-blinded, observational study examined the use and diagnostic impact of two Electronic Health Record (EHR) auto-populated QTc values on ECG reports, QTcB and QTcFri. ECG reports were collected from May 2019 to April 2020 at Cape Fear Valley Medical Center - a suburban, tertiary care center in Fayetteville, North Carolina. ECGs were recorded using Phillips IntelliSpace ECG devices (Phillips Healthcare Solutions, Franklin, TN). Standard 12‐lead ECGs were collected with 25 mm/s paper speed, 10 mm/1 mV amplitude, and 250 Hz sampling rate. Gathered data from Epic EHR ECG reports (Epic Systems Corporation, Verona, WI) included patient age, sex, HR, QRS duration (QRSd), QT interval, QTcB, and QTcFri. The study was approved by the Cape Fear Valley Health Internal Review Board and data were collected retrospectively and deidentified. 

Patient population

ECG reports were obtained in the hospital from both male and female patients aging from 18 to 105 years. ECG reports were assessed for a minimum heart rate of 60 bpm, a maximum heart rate of 120 bpm, and a QRS duration of less than 120 ms. ECG report data were inspected for quality and excluded from the analysis if HR < 60 bpm or > 120 bpm, QRS > 120 ms, missing EHR parameters, or if erroneous in the extreme (e.g. age 120 years).

The study was designed with the intent of applying minimal exclusion criteria to maximize the study sample size while ensuring the fidelity of the data. The goal of the sample was for extrapolation to generalized hospital populations. ECG reports used in the study came from across the health system: Outpatient Surgery, Emergency Department, Med-Surgical Floors, Post-Anesthesia Care Units, the Intensive Care Unit, etc.

QTc analysis

QTcB was calculated using Phillips IntelliSpace ECG devices (Phillips Healthcare). QTcFri was calculated using the Fridericia formula (Table 1).

**Table 1:** Common QTc formulae from 1920 – 2014 QTc – Rate corrected QT interval, QT – Q wave to T wave interval, RR – R wave to R wave interval, HR – heart rate

Creator	QTcX	Year	Formula
Bazett [9]	QTcB	1920	QTc = QT/√RR
Fridericia [10]	QTcFri	1920	QTc =QT/^3^√RR
Hodges [11]	QTcH	1983	QTc =QT + 1.75(HR-60)
Frammingham [12]	QTcFra	1992	QTc = QT + 0.154(1-RR)
Dmitrienke [13]	QTcD	2005	QTc = QT/RR^0.413^
Rautaharju [6]	QTcF	2014	QTc = QT − 185 (HR/60 − 1) + k (k = 6 ms [male], 0 ms [female])

Data were analyzed according to gender and for the aggregate total study population. General statistical methods included range, median, mean, and standard deviation. Confidence intervals were assessed to maintain the fidelity of analysis. The normality of data distribution was assessed with Kolmogorov-Smirnov testing. The Wilcoxon rank-sum test was then performed to confirm a statistically significant difference between the Bazett and Fridericia formulae. A value of p<0.05 was interpreted as significant. Statistical analysis was performed using SPSS statistical software (IBM Statistics, v. 26; IBM Corp, Armonk, NY).

QT interval rate correction was calculated by the Epic EHR and documented on each ECG report. Data were then stratified based on the presence of prolonged QTc, 450 and 460 ms standard cutoffs for men and women, respectively, and QTc greater than 500 ms - indicating greater risk for ventricular arrhythmia. The differences between the two formulae and their magnitudes were recorded along with respective means, standard deviations, and confidence intervals.

## Results

General characteristics

A total of 61,946 ECG tracings were performed from May 2019 to April 2020 and uploaded to the Epic EHR at Cape Fear Valley Medical Center. After selecting the tracings that met the inclusion criteria, 44,566 ECGs were evaluated in this study (Table 2).

**Table 2:** ECG data obtained at Cape Fear Valley Medical Center from May 2019 to April 2020 ECG: electrocardiogram

Men (n=19160)						99% CI	
	Mean	Standard Deviation	Minimum	Maximum	Median	Lower	Upper
Age (years)	57	16.3	18	103	59	57.0	57.6
Heart Rate (bpm)	83	14.6	61	120	82	83.2	87.7
QRS Duration (ms)	95	10.6	49	119	94	94.6	95.0
QT Interval (ms)	383	40.8	232	698	381	383.1	384.7
QTc Bazett (ms)	448	37.9	303	749	444	447.7	449.1
QTc Fridericia (ms)	425	35.3	277	730	421	424.8	426.1
∆QTc (ms)	23	12.8	1	78	22	22.8	23.2
Women (n=25406)							
Age (years)	57	18.3	18	105	58	56.7	57.3
Heart Rate (bpm)	83	14.6	60	120	83	82.7	83.2
QRS Duration (ms)	91	10.6	43	119	91	91.1	91.5
QT Interval (ms)	388	42.3	183	695	386	388.2	389.6
QTc Bazett (ms)	453	37.6	209	738	448	452.0	453.2
QTc Fridericia (ms)	430	35.7	200	716	425	429.3	430.5
∆QTc (ms)	22.7	12.9	0	72	22	22.5	22.9
Total (n=44566)							
Age (years)	57	17.5	18	105	59	56.9	57.3
Heart Rate (bpm)	83	14.7	60	120	81	83.0	83.3
QRS Duration (ms)	92.8	10.7	43	119	92	92.7	92.9
QT Interval (ms)	387	41.7	183	698	384	386.2	387.2
QTc Bazett (ms)	451	37.8	209	749	446	450.3	451.3
QTc Fridericia (ms)	428	35.6	200	730	423	427.5	428.4
∆QTc (ms)	22.8	12.9	0	78	22	22.7	23.0

**Figure 1:**
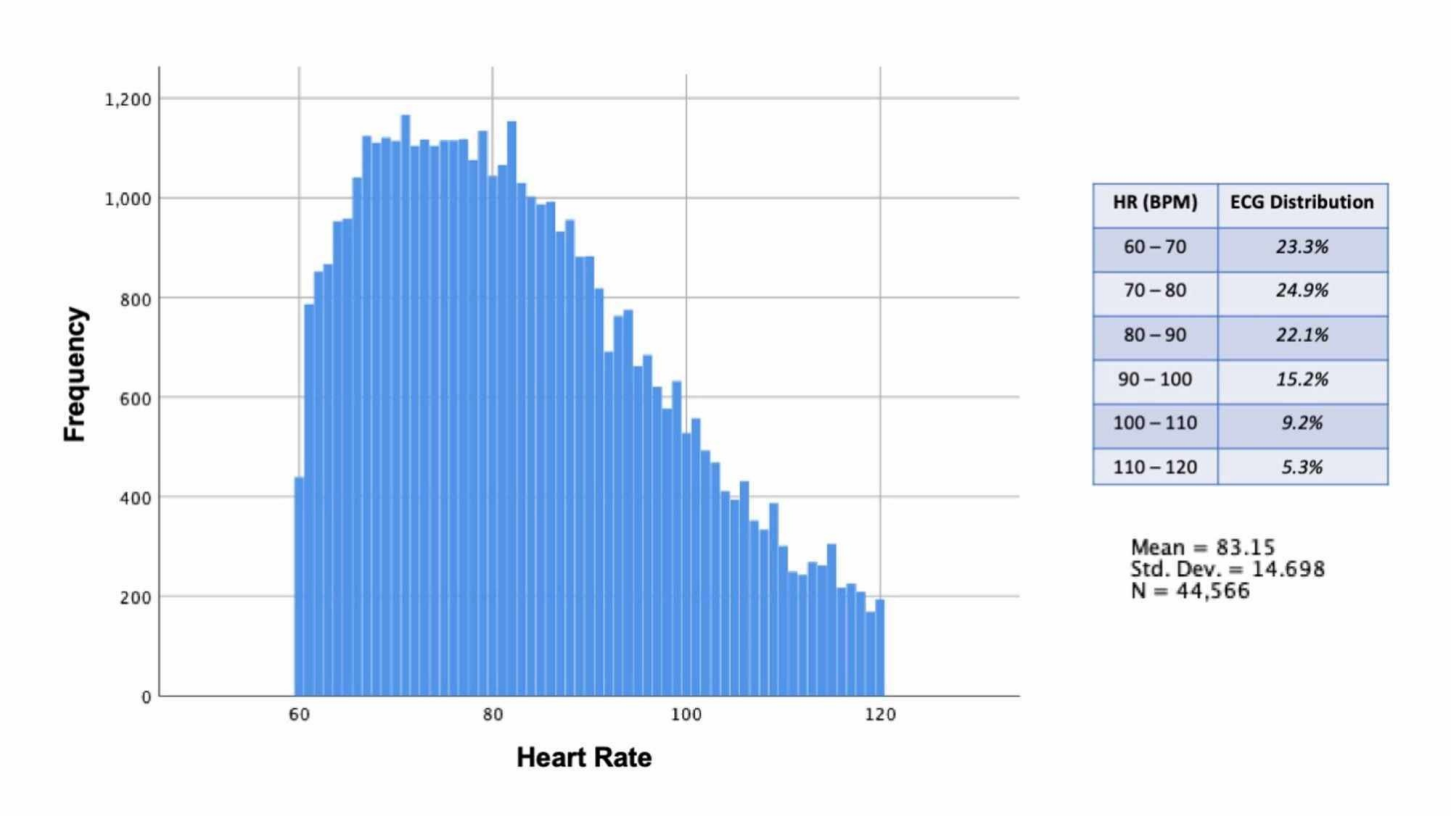
Electrocardiogram report heart rate distribution *Created with IBM SPSS statistical software (IBM Corp., Armonk, NY)

The data showed 57% of the tracings were performed on females and the average age for both genders was 57 ± 17.5 years. The mean heart rate was 83 ± 14.7 bpm and the study population distribution is shown in Figure 1.

The mean difference between the Bazett and Fridericia formulae for the total study population demonstrated a decrease in calculated QTc of 23 ± 12.9 ms (99% CI: 22.7, 23.0 ms). These data show minimal variation between sexes with mean age, heart rate, uncorrected QT, QTcB, QTcFri, and ∆QTc varying by less than 2%. Mean QRSd showed the most variation between sexes with male QRSd 95 ± 10.6 ms. (99% CI: 94.6 ms, 95.0 ms) and female QRSd 91 ± 10.6 ms. (99% CI: 91.1 ms, 91.5 ms).

QTc formula comparison

Nonparametric distribution of the data was confirmed by Kolmogorov-Smirnov testing: D_Bazett_ (44,566) = 0.076, p = <0.0001 and D_Fridericia_ (44,566) = 0.081, p = <0.0001.

Wilcoxon rank-sum testing (Table 3) of the difference (QTcB - QTcFri) revealed 44,127 ranks with a negative difference, 0 ranks with a positive difference, and 439 ties, p <0.001 (99% CI: 22.5 ms, 23.0 ms).

**Table 3:** QTc comparison statistical analysis

Wilcoxon Rank Sum Test QTcB-QTcF		
n	44,127	Negative Difference: 44,127
p	< 0.001	Positive Difference: 0
Difference	22.5 ms	Tie: 439
99% CI (ms)	22.5, 23.0	

Prolonged QTc group analysis

QT interval prolongation criteria were met in 37.4% of ECG reports (Table 4). Male ECG reports showed an increased incidence of QT interval prolongation but less frequent incidence than females at severely prolonged QTC > 500 ms. Prolonged QT intervals corrected to the normal QT interval in 56.2% of cases with QTcFri (Table 4).

**Table 4:** Prolonged QTc subgroup analysis

	Prolonged QTcB		Prolonged QTcFri		Corrected to Normal via QTcFri		∆QTc (ms)[(99% CI)
Women ( >460 ms)	34.20%	8684/25406	14.60%	3714/25406	57.20%	4970/8684	27.5 (27.2, 27.9)
Men ( >450 ms)	41.70%	7981/19160	18.70%	3580/19160	55.10%	4401/7981	27.7 (27.3, 28.1)
Total	37.40%	16665/44566	19.60%	8754/44566	56.20%	9371/16665	27.6 (27.3, 27.9)
Mean (ms) (99% CI)	487 (486, 488)		459 (459, 460)		-	-	27.6 (27.3, 27.9)
Median (ms)	477		452		-	-	28
Standard Deviation (ms)	33.7		34.5		-	-	13.2

Utilizing QTcFri in place of QTcB decreased the corrected QT interval duration misinterpretation by 21% or 9,371 cases. The mean decrease in corrected QT interval duration was 28 ± 13.2 ms (99% CI: 27.3 ms, 27.9 ms).

Table 5 shows that QTcFri use reduced the number of ECG reports with QTc > 500 ms by 57.3%. A total of 125 ECG reports, 117 females and eight males, demonstrated correction to normal gender-specific QTc with QTcFri. The mean decreased in QTc with the Fridericia formula when QTcB > 500 ms was 31 ± 14.5 ms (99% CI: 30.4, 31.7).

**Table 5:** Prolonged QTc > 500 ms subgroup analysis

	QTcB >500 ms		QTcFri >500 ms		Corrected to QTcFri < 500 ms		∆QTc (ms) (99% CI)
								Women	8.60%	2185/25406	3.80%	975/25406	55.30%	1210/2185	30.6 (29.8, 31.4)
Men	7.70%	1465/19160	3.50%	665/19160	54.60%	801/1465	31.8 (30.8, 32.7)
Total	8.20%	3650/44566	3.50%	1559/44566	57.30%	2901/3650	31.0 (30.4, 31.7)
Mean (ms) (99% CI)	536 (535, 538)		505 (504, 507)		-	-	31.0 (30.4, 31.7)
Median (ms)	523		494		-	-	32
Standard Deviation (ms)	38.3		41.1		-	-	14.5

## Discussion

Study design

This study was undertaken to demonstrate the availability of evidence-directed QTc formulae on ECG reports without additional calculation. The Fridericia formula was not chosen as an alternative for the Bazett formula on the Epic EHR system, but QTcFri was programmed in the ECG report algorithm when the EHR was installed in May 2019. ECG reports were accessed for the parameters above which constituted the majority of the clinically relevant information found on said reports. The ECG reports did not comment on the computer interpretation of ECG rhythm or diagnosis.

This study did not take heart rhythm into account, QT-interval prolonging medications, electrolyte abnormalities present at the time of the electrocardiographic study, or medical history. Inclusion criteria were created to minimize the impact of these unknown factors. The physiologic heart rate without prolonged interventricular conduction delay was selected as a key criterion to remove, as much as possible, data interference from bradyarrhythmias and tachyarrhythmias.

Main findings

The results of the Wilcoxon rank-sum analysis, with a p-value of <0.001, confirms a statistically significant difference between the two formulae. In examining 44,566 ECG reports, when a difference existed, the magnitude was on average 22 ms but extended up to a maximum of 78 ms. This analysis of rate-corrected QT evaluation using two power function formulae corroborates the 2009 American Heart Association (AHA)/American College of Cardiology Foundation (ACCF)/Heart Rhythm Society (HRS) recommendation to avoid QTcB use.

The greatest benefit of utilizing non-QTcB rate correction was evident when looking at the prolonged and severely prolonged QTc subgroups. The 16,665 ECG reports with prolonged QTcB corrected to normal QTc in 56% of cases when the Fridericia formula was applied. Evaluation of the 44,566 ECG reports in our health system over a one-year period showed 21% of the total ECGs corrected to normal QTc with evidence-based formulae. Inaccurately documenting one in five patient test results in the medical record is compelling evidence to change the standard of measurement.

The Fridericia formula is a power function; therefore, it is not endorsed by the AHA/ACCF/HRS guidelines. While QTcFri shows greater rate dependency than the Rautaharju formula, QTcFri also shows less rate dependency than the Bazett formula [2]. The Fridericia formula has been demonstrated in research to have a clinical benefit at physiologic heart rates [5]. QTcFri is the only calculation besides the Bazett formula mentioned by name in the US Food and Drug Administration’s guidance for clinical trial evaluation of medication-induced QT interval prolongation [14].

Clinical implications

QT interval correction affects clinical decision-making. This data presents the opportunity to better educate providers on accurate methods to assess the QT interval. Viskin et al. (2005) demonstrated that the non-arrhythmia specialists (General Cardiologists, Internists, Neurologists, Pediatricians, Emergency Medicine Physicians, Intensivists, and Gastroenterologists) are not well-trained in measuring QT intervals, calculating QT intervals, or identifying QT interval prolongation [15]. Many providers are also not aware of which formula is used on ECGs or that multiple formulae exist. Studies like Viskin et al. (2005), in conjunction with the data above, demonstrate the clinical need for improved education to improve patient care [15].

When the QTc on ECG is interpreted as prolonged, therapies are potentially withheld or discontinued. This area of research holds promise for improving patient care: regarding the patient length of stay and nosocomial infection risk. With the demand for the use of QT-prolonging medications currently on the rise, utilizing more accurate data in the treatment of patients is needed.

Currently, the Epic EHR possesses the functionality to calculate QTcFri based on uploaded ECG data and to compare this calculation with ECG-documented QTcB data on each ECG report. This EHR capability creates the potential to increase provider awareness of evidence-based QTc formula and their integration into clinical practice.

Limitations and future research

This project was a single-center, non-randomized, non-blinded, retrospective, observational analysis, all of which has the potential to bias the data and subsequent results. These factors must be taken into consideration when evaluating the data.

ECG rhythm could not be determined from Epic ECG reports. The presence of atrial fibrillation, atrial flutter, and ventricular arrhythmia could not be assessed. Physiologic heart rate (60-120 bpm) was selected as an inclusion criterion to minimize the impact of arrhythmias on the fidelity of the data. The exclusion extent of irregular rhythms cannot be confirmed without viewing each of the 44,566 ECG tracings. Also, screening of cardiovascular disease history or QT-prolonging medication administration was not performed to allow a heterogeneous, real-world evaluation of patients independent of presentation.

Lastly, the fact that EHR systems possess the functionality to encode additional formulae like Fridericia into ECG reports presents the opportunity to employ guideline-based QTc functions like Hodges’ formula (1983) or Rautaharju formula (2014) - both linear functions endorsed by the AHA/ACCF/HRS. The QTcR formula also has the added benefit of gender specificity evaluation for risk of TdP, with an adjusted QTc threshold for increased risk for ventricular arrhythmia at 477 ms [16]. Recent research like Rabkin et al. (2015) demonstrates that the evaluation of this data and prospective studies with linear-function formulae may provide greater accuracy and further clinical benefit [2].

## Conclusions

EHR QTc analysis with QTcFri demonstrated a mean decrease of 22 ms over 44,566 ECG tracings obtained at a single center between May 2019 and April 2020. During this time, 9,371 (21%) of ECGs performed in a tertiary care facility were inaccurately documented with prolonged QTc. Accurate QT interval calculation will decrease the inappropriately documented test results and better inform downstream clinical decision-making through a wider scope of therapeutic intervention. This analysis is readily available to clinicians without any calculation required, and awareness of its existence will improve patient care. Just as the Fridericia formula was programmed in our EHR, the most accurate and guideline-directed QTc formulae are able to be employed. The utilization of this resource will provide the most accurate and clinically relevant data to inform clinical decision-making. EHR capability to program evidence-based QTc formulae without provider computation also merits further research to aid in the establishment of a new standard of QT rate correction.
